# Review of Aluminum Alloy Development for Wire Arc Additive Manufacturing

**DOI:** 10.3390/ma14185370

**Published:** 2021-09-17

**Authors:** Geir Langelandsvik, Odd M. Akselsen, Trond Furu, Hans J. Roven

**Affiliations:** 1 SINTEF Industry, Richard Birkelands veg 2B, 7034 Trondheim, Norway; odd.m.akselsen@sintef.no; 2 Norsk Hydro, Corporate R&D Headquarter, 0283 Oslo, Norway; trond.furu@hydro.com; 3 Department of Materials Science and Engineering, NTNU Norwegian University of Science and Technology, 7034 Trondheim, Norway; hans.j.roven@ntnu.no

**Keywords:** wire arc additive manufacturing, WAAM, additive manufacturing, aluminum, columnar-equiaxed-transition, alloy development, light alloys

## Abstract

Processing of aluminum alloys by wire arc additive manufacturing (WAAM) gained significant attention from industry and academia in the last decade. With the possibility to create large and relatively complex parts at low investment and operational expenses, WAAM is well-suited for implementation in a range of industries. The process nature involves fusion melting of a feedstock wire by an electric arc where metal droplets are strategically deposited in a layer-by-layer fashion to create the final shape. The inherent fusion and solidification characteristics in WAAM are governing several aspects of the final material, herein process-related defects such as porosity and cracking, microstructure, properties, and performance. Coupled to all mentioned aspects is the alloy composition, which at present is highly restricted for WAAM of aluminum but received considerable attention in later years. This review article describes common quality issues related to WAAM of aluminum, i.e., porosity, residual stresses, and cracking. Measures to combat these challenges are further outlined, with special attention to the alloy composition. The state-of-the-art of aluminum alloy selection and measures to further enhance the performance of aluminum WAAM materials are presented. Strategies for further development of new alloys are discussed, with attention on the importance of reducing crack susceptibility and grain refinement.

## 1. Wire Arc Additive Manufacturing

The ASTM standard F2792 defines additive manufacturing (AM) as *“The process of joining materials to make objects from 3D model data, usually layer-upon-layer, as opposed to subtractive manufacturing methodologies”* [1]. AM is hence regarded as a bottom-up technology as it creates a shape from zero, in contrast to carving from a block material or casting into a preform. AM is capable of depositing metals, ceramics, polymers, and composites by a wide range of different technologies. Metals and alloys are usually melted by a fusion heat source and deposited on a build substrate. The metal feedstock may be in the form of powder or wire, and available heat sources are laser, electron beam, plasma, and electric arc. Comprehensive reviews of AM of metals are available in the open literature [2,3,4].

Layer-by-layer fusion of metallic wires by an electric arc is termed wire arc additive manufacturing (WAAM). A WAAM assembly consists of several constituents. The feedstock for deposition is in the form of a wire. This material is melted by a heat source, i.e., the electric arc. Plasma arc welding (PAW) and gas tungsten metal arc welding (GTAW), as well as gas metal arc welding (GMAW) with its variants, are common fusion sources for WAAM. The arc torch is connected to a welding apparatus for parameter control. Further, the torch is mounted to a manipulator system, e.g., a robotic arm, or computer numerical controlled (CNC) table. The manipulator system provides strategic displacement of the torch for layer-wise deposition. The material is deposited on a substrate, commonly a metal plate.

WAAM exhibits attractive qualities for wide-spread use throughout several industrial sectors. The inherent advantages of high deposition rate and virtually unlimited build envelope enable production of larger structures with short lead times. The low investment cost of WAAM is an enabling factor for small and medium-sized enterprises to utilize AM to shorten the supply chain from production to operation.

Series production of metallic components by WAAM is suitable for most industries. However, some sectors are to date less appropriate than others. For instance, the automotive industry produces parts in large quantities with a constant focus on reducing cycle time. WAAM is currently not efficient and profitable for large volume series production for automotive vehicles. However, WAAM can be suitable for prototyping of new automotive parts as a successor of rapid prototyping [5]. In applications where performance is of utmost importance, e.g., in motorsports, racing, and luxury car segments, WAAM is highly appropriate.

Several researchers pointed out the aerospace and aeronautic sectors as potential markets for WAAM [6,7,8]. The annual production volumes are relatively low, which is beneficial for WAAM. Weight reduction to suppress fuel consumption is highly important in these sectors. This can be obtained by improved designs not attainable by milling which enhance strength and fatigue resistance. This can again bring about slimmer and lighter structures through topology optimization [9]. Several airplane parts are today manufactured by milling from billets with cut-off ratios surpassing 90% [6]. Material waste, and hence, cost, can be greatly reduced by combining near-net shaping by WAAM and final surface milling. Given the fact that aerospace and aeronautic industries utilize high-cost, high-quality materials, the cost savings can be massive. Similar cost savings can be obtained in energy and nuclear industries of high-temperature resistant materials and in production of fans and impellers for electronic applications [10,11]. The marine industry faces increased competition and lower margins, pushing the adaption of additive manufacturing into design and production. In this context, WAAM is regarded as being highly suitable for production of large, complex structures such as bulbous bows, rudders, and ship propellers [12].

WAAM has the potential to perform maintenance, repair, and spare part production, although these possibilities have been less communicated. The possibility to maintain and repair components instead of scrapping them induces major benefits in terms of cost and environmental impact. Turbine blades were projected as a suitable recipient for WAAM maintenance [13]. The Norwegian energy company Equinor initiated pipeline maintenance with their WAAM system Weldar [14]. WAAM and AM can disrupt the supply chain and storage of spare parts. Sectors vulnerable for operational downtime, e.g., marine, offshore oil&gas and defense, are dependent on the rapid delivery of spare parts. The solution today is extensive warehousing of spare parts. By shifting the focus from a ‘just-in-case’ to a ‘just-in-time’ philosophy, spare part warehousing may be eliminated. In summary, operational down time, warehouse storage, and scrapping can be greatly reduced by proper implementation of AM and WAAM into the industry.

WAAM is a versatile and low-cost technology compared to that of other AM processes. Components can be made with a deposition rate up to several kg/h for aluminum, which is orders of magnitude higher than that of powder based processes [15,16]. An electric arc as heat source enables localized inert gas shielding; hence, inert build chambers used for powder-and-laser AM can be omitted. The exception is reactive metals such as titanium and Al-Li alloys, which require an inert build chamber. Localized gas shielding reduces the cost and enable unlimited build volumes. The hardware needed for WAAM (robotic arm, weld power source, feedstock wire) is relatively cheap compared to that of laser- and electron-beam systems. Hardware can be bought ‘off the shelf’ from different suppliers and tailored to the configuration needed.

The use of wire feedstock for deposition brings several benefits. Wires are cheaper than powder feedstock and are easier to store, transport, and handle. Powder feedstock may also induce explosion hazard and impact on biological life. The energy efficiency of metal arc deposition is high. Up to 90% of the energy is supplied to the workpiece [17]. This is in strong contrast to laser- and electron-beam systems, where 2–20% of the energy is utilized for deposition of aluminum alloys [18,19]. In conclusion, WAAM is especially appropriate for production of large-volume parts made by costly materials where scrap metal savings are important. As an example, Williams et al. [6] introduced material savings close to 500 kg per part for aluminum wing ribs by WAAM compared to that of subtractive machining. Bekker et al. [20] performed a life-cycle assessment (LCA) comparing the environmental impact of CNC milling, green sand casting, and WAAM of 308L stainless steel from cradle to gate. Sand casting and WAAM performed equally well in terms of environmental impact, while outrunning CNC milling. However, the conclusions were shown to be vulnerable to the selection of material and material waste from milling. Guo developed a cost and carbon footprint assessment model for WAAM [21]. For low production volumes with high complexity, WAAM was shown to be superior to casting and CNC milling in terms of cost and greenhouse gas emissions. Thus, WAAM is considered a relatively green manufacturing route for specific applications.

## 2. Aluminum Alloys

Aluminum is the most abundant metallic element in the Earth’s crust. The metal possesses a range of desired properties, such as low weight, high specific strength, good thermal and electrical conductivity, and excellent corrosion resistance and formability. Pure aluminum has a density of 2.7 $g/cm^{3}$ (one third of the density of steel) and is workable due to the inherent face centered cubic (FCC) crystal structure with low resistance for dislocation slip. The excellent corrosion resistance in oxidizing environments is due to an instantaneous formation of a dense, self-restoring oxide film.

Properties such as strength, weldability, and formability of aluminum can be tailored for specific applications through addition of alloying elements. Common solutes to aluminum are copper, manganese, silicon, magnesium, and zinc. Aluminum alloys are divided into two classes, i.e., wrought and cast alloys. The latter category is heavily alloyed for improved castability and to obtain reasonable strength, and is seldom utilized as wire consumables for arc deposition. Thus, wrought alloys are covered henceforth. Aluminum wrought alloys are divided into eight categories, called series, based on their main alloying elements. A short description of the series with their properties related to WAAM are given in the following.

1000-series are commonly referred to as commercial pure aluminum with presence of less than 1% of other elements. The minor presence of solute is often impurity elements from the primary production process, such as iron and silicon. Commercial pure aluminum finds no structural applications due to its softness. Thus, alloys from this series are seldom utilized for WAAM. However, due to the excellent corrosion properties, the alloy series may find applicability as cladding material.

2000-series aluminum-copper alloys possess high strength but poor corrosion resistance. The sound mechanical properties originate from atomic clusters (GP zones) and plate-shaped Al${}_{2}$Cu (θ′ and θ″) precipitates formed during heat treatment. Wire consumables of the 2319 alloy is commercially available, originally developed for joining with 2219 plates and forgings. The 2319 and 2219 alloys are well-suited for WAAM, as demonstrated by several authors [22,23,24,25,26,27,28,29] together with the fatigue-resistant 2024 alloy [30,31].

3000-series contain manganese as main alloying element. These alloys are all-purpose alloys for, e.g., beverage cans with intermediate strength levels and good formability. 3000-alloys were never processed by WAAM, and seldom utilized as filler for welding applications [32].

4000-series aluminum alloys possess intermediate strength levels from solid solution strengthening of silicon. Al-Si alloys are mostly used for casting purposes due to excellent flowability and decreased thermal shrinkage. For wrought applications, welding and brazing applications are most widespread. 4043 is the workhorse for arc welding and is used in a range of industries due to the excellent weldability towards a wide range of aluminum alloys. 4043 is consequently heavily studied for WAAM [33,34,35,36,37,38]. The high-silicon brazing alloy 4047 was also demonstrated for WAAM applications [39,40,41].

5000-series aluminum alloys have magnesium as main alloying element and trace amounts of manganese for dispersion strengthening. The magnesium content increases the corrosion resistance in marine environments and is commonly used in shipbuilding. The strain hardening response of Al-Mg alloys is significant due to the Portevin–Le Chatelier effect [42]. The excellent properties of 5000-alloys in WAAM materials were frequently demonstrated in later years, including 5087 [26,43], 5183 [44,45,46,47] and 5356 [48,49,50] with commercial feedstock.

6000-series Al-Mg-Si alloys are heat-treatable through precipitation of semicoherent Mg${}_{2}$Si $\beta^{\prime }$ and $\beta^{\prime \prime }$-precipitates for increased strength. These alloys have a wide range of applications, especially as extruded profiles. The high cracking susceptibility of 6000-series alloys during solidification makes these alloys difficult to process by additive manufacturing [51]. Thus, commercial feedstock for WAAM and welding is scarcely available. To the authors knowledge, the only Al-Mg-Si alloy available as weld filler is 6063 [52].

7000-series aluminum alloys have zinc as main alloying element, together with balanced additions of copper and magnesium. Such alloys obtain remarkable mechanical properties through precipitation of MgZn${}_{2}$ and Mg(Zn,Cu,Al)${}_{2}$η-phases during artificial heat treatment. In fact, the 7068 alloy is the strongest commercially available aluminum alloy, and is commonly utilized for military aircraft applications [53]. 7000-series alloys are to date not available as commercial feedstock for WAAM.

## 3. Challenges Related to Aluminium WAAM

The mechanical properties of aluminum alloys fabricated by WAAM show comparable and even superior performance compared to that of their wrought counterpart [54]. However, WAAM parts exhibit defects, which limits their applicability in industrial service and even the extent of available alloys. These challenges are related to the process stability (path planning, parameter setup, melt pool shielding, etc.) and alloy chemistry. The most prominent challenges related to WAAM of aluminum are covered in this section, namely porosity formation, residual stresses, and cracking.

### 3.1. Porosity

Cavities are the most common defect in aluminum alloys processed by WAAM. The cavities may be in the form of shrinkage pores or metallurgical porosity. The type of void can be separated by their sphericity. Metallurgical pores have a sphericity close to 1, while shrinkage pores have significantly lower values than 1 [55]. Shrinkage pores are formed due to the large thermal shrinkage of aluminum upon cooling (∼23 μm/mK) and the difficulties in ‘back-filling’ the cavity. Such defects are irregularly shaped, and they are often found in vicinity of primary phases such as alpha-aluminum dendrites. The shrinkage may also result in cracking, as covered in Section 3.3 of this review.

Vaporization of metallic elements during WAAM is a little discussed topic in literature. Elements with high vapor pressure like Mg, Zn, and Li vaporize at elevated temperature and leave porosity in the structure [56,57]. Up to 20% magnesium loss in 5000-alloys was reported for WAAM, dependent on the supplied heat input [58]. However, the consequence of elemental losses on mechanical properties received more attention than the correlated porosity formation.

The most common cavity defect related to WAAM of aluminum is hydrogen porosity. This defect is spherical in shape and is formed due to precipitation of supersaturated hydrogen during the solidification stage. The large solubility difference of hydrogen in liquid and solid state of aluminum is responsible for the ease of porosity formation [59]. The solid-state solubility of hydrogen in aluminum is virtually nonexistent, but it can be slightly increased by lithium in solid solution, and at dislocations, vacancies and certain precipitates [60].

Due to the low solid-state hydrogen solubility, the remaining liquid during solidification is enriched in hydrogen, which may supersede the solubility limit at a given temperature. The pores are believed to form by nucleation and growth in the liquid, and the topic was extensively researched and modeled [61]. Upon supersaturation, nucleation of a pore is initiated when the gas pressure in the melt pool overcomes the combined local fluid pressure and the surface tension of forming gaseous species [62]. Further pore growth is governed by diffusion, as modeled by Li and Chang [63]. In WAAM, the formed pore either floats to the top of the melt pool by buoyancy or is trapped by the advancing solidification front. A small fraction of hydrogen can also exist in supersaturated state due to the rapid solidification in WAAM [64]. This hydrogen can be precipitated as secondary pores under postdeposition heat treatments [65].

The presence of hydrogen is a prerequisite for forming porosity. There are numerous sources to how hydrogen can be introduced to the final aluminum WAAM structure. The internal hydrogen content is governed by the refining processes of the primary metal in the casthouse, and any reaction between water, moisture, and hydrated oxide films with aluminum. Further, the produced wire can adsorb moisture on its surface during storage. Hydrocarbons in the form of grit, oil, or paint on the wire surface play a major role in increasing the hydrogen content. At last, the hydrogen level in the shield gas and moisture pickup from air during arc deposition induce hydrogen porosity.

Ryan et al. [66] proposed hydrocarbon contamination on the wire feedstock surface as a considerable hydrogen source. Wires with poor surface quality trap grit and moisture in cavities and cracks, which becomes free hydrogen when the wire is melted by an electric arc. Aluminum is reactive with oxygen and humidity in air, even at room temperature. Aluminum instantly produces a thin Al${}_{2}$O${}_{3}$ layer in contact with oxygen. The oxide layer is further hydrated in contact with the humidity in air, creating an amorphous Al_2_O_3_ · 3H${}_{2}$O phase [67]. Physisorbed and chemisorbed water molecules on the feedstock wire decompose during WAAM into atomic hydrogen. This hydrogen source can be eliminated by thermal treatment (decomposition of the hydrated layer) or by laser cleaning [68]. In-situ resistance heating of the consumable wire shortly before deposition showed promising results with reducing the porosity content in aluminum alloys [30].

### 3.2. Residual Stresses and Distortion

AM methods which involve melting of the input materials induce residual stresses in the material. The high energy fusion source (e.g., arc, laser) rapidly heats the feedstock material, thus expanding the material. Further, the feedstock is deposited and allowed to solidify and cool. In the case of aluminum, cooling results in contraction of the material. Because of the uneven solidification and cooling rate of the material, the deposited material experiences uneven contraction [69]. Constraints from the base plate and already solidified material deny the last portion of material to contract, in which residual stresses are developed. The stresses are usually tensile in nature, which lowers the fatigue resistance of the whole component [70]. If the residual stress levels surpass the yield strength, or the constraint (e.g., base plate) is removed, the material distorts. Distortions are generally unwanted, as the geometrical accuracy of the component is aggravated. Prominent actions to reduce distortions are parameter selection (heat input and dwell times), path planning, and auxiliary processes such as heat treatment and high-impact rolling [70,71]. As residual stresses were shown to be linearly related to the material hardness of quenched aluminum alloys [72], the residual stresses are to some degree dependent on alloy composition.

### 3.3. Cracking

Joining of materials by fusion-based methods can imply a range of cracking mechanisms in the as-solidified weld. Hot cracking in the partial melted zone is a common cracking mechanism in arc welding, but it was not reported for WAAM materials [73]. The most prominent mechanism for aluminum and its alloys in WAAM is solidification cracking, which happens in the liquid melt pool. Cracks formed are intergranular and irremediable when first formed. Solidification cracking was heavily studied for aluminum castings [74].

**Figure 1:**
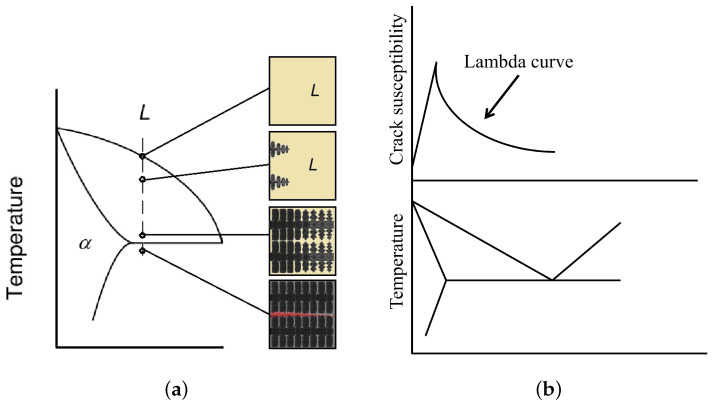
Solidification cracking in materials as a consequence of microstructure and solidification range. (**a**) Solidification regime in two-phase region of aluminum with a columnar dendritic structure. An intergranular crack is shown in lower microstructure, marked in red. (**b**) Solidification crack susceptibility as a function of alloy composition, and solidification range for an arbitrary binary eutectic alloy. Reproduced with permission from Song et al., Journal of Magnesium and Alloys; published by Elsevier, 2016 [76].

The main factors affecting the solidification cracking susceptibility are related to the grain morphology and solidification characteristics [75]. It is generally accepted that a wide solidification range increases the crack susceptibility. A large difference between the liquidus and the solidus temperature results in the formation of a well-established solid network of primary phase where the remaining liquid has limited mobility close to solidus. The cracking susceptibility is further enhanced when the primary metal solidifies in a columnar dendritic manner. The mechanism is shown in Figure 1a. Close to the solidus temperature, isolated liquid pockets strain the solid network due to shrinkage. As remaining liquid is unable to ‘back-fill’ the pockets, tensile stresses are exerted on the solid network. Cracking occurs if the tensile stresses exceed the tensile strength of the material at the given temperature.

The two-phase temperature range is closely related to alloy composition. As shown in Figure 1b for an arbitrary binary eutectic system, the cracking susceptibility increases toward compositions with longer solidification ranges. This correlation is commonly called the lambda curve [76]. Solidification cracking can therefore be depressed by proper alloy selection. The thermal shrinkage and liquid viscosity of alloying elements are also important factors for solidification cracking. The fact that silicon decreases the viscosity (i.e., better flowability) and expands upon cooling contributes to the low-crack susceptibility of Al-Si alloys.

Several theories and models were developed to predict the solidification crack susceptibility of metals, and in particular, aluminum alloys. One of the first well-predicting models was presented by Clyne and Davies [77], which considered the fraction of time where the alloy was susceptible to cracking. The resulting crack susceptibility coefficient CSC is presented in Equation (1). $t_{V}$ refers to the time when the solid fraction during solidification is between 90–99%, which is the vulnerable stage where the solid network of the metal can disrupt and produce solidification cracks. $t_{R}$ is the so-called relaxation time where the alloy can redistribute liquid without constraint, usually between 60–90% fraction of solid. The CSC criterion is well-suited for development of Lambda-curves, as presented in Figure 1b.

(1) $$CSC=\frac{t_{V}}{t_{R}}$$

A more advanced approach to assess the cracking susceptibility was presented by Rappaz et al. [78] through the Rappaz–Drezet–Gremaud (RDG) criterion. Instead of time as basis for crack susceptibility, the mass balance and a critical strain rate were considered. This involves the liquid flow at interdendritic areas to accommodate shrinkage and deformation of the solid network of dendrites. The criterion is based on the initiation of a void in the network, which grows when first formed into a crack. If the local pressure supersedes a critical pressure, the void is created. The hot crack sensitivity (HCS) is a function of the deformation rate in the solid phase $\dot{\epsilon_{p}}$, Equation (2). The risk of cracking increases with increasing value of HCS.

(2) $$HCS=1/\dot{\epsilon_{p}}$$

where factors like shrinkage β, solidification front velocity $v_{T}$, liquid flowability $v_{l}$ and fraction solid and liquid ($f_{s}$ and $f_{l}$, respectively) are taken into account, Equation (3) [74,78].

(3) $$\frac{df_{l}v_{l}}{dx}+\left(1+\beta \right)f_{s}\dot{\epsilon_{p}}-v_{T}\beta \frac{df_{s}}{dx}=0$$

## 4. Strategies to Mitigate Defects in WAAM

The mentioned challenges and defects related to WAAM of aluminum restrict the mechanical properties and integrity of finished components. A wide range of measures to mitigate these defects was proposed in the open literature. Although well-covered in other comprehensive reviews, a short summary of proposed actions to enhance the quality of WAAM components is given in the following.

### 4.1. Mechanical Impact

Several researchers examined the effect of striking the deposited layer with mechanical forces to improve the material quality. Applying high pressure to every deposited layer was shown to be efficient in pore closure and strength enhancement. The enhanced strength is a result of grain refinement through recrystallization, enhanced dislocation density, and dispersion of interdendritic phases. Notable strategies are postlayer rolling [79], demonstrated for alloys such as 2319 [26] and 5087 [43]; pneumatic hammering [80]; hot isostatic pressing [81]; laser shock peening [82], and ultrasonic peening [83]. All demonstration cases were performed on straight walls with one layer of thickness; the applicability of each auxiliary process needs to be assessed for more complex designs. In addition, the investment and operational costs need to be considered. An interlayer process decreases the overall production efficiency and affects the total cost in production.

### 4.2. Hardware

Considerable efforts were devoted to assessing the most suitable hardware for successful and high-quality WAAM. Deposition torches, shield gas composition, and substrate tables were of special interest. The two common deposition principles for WAAM are based on gas tungsten arc welding (GTAW) and gas metal arc welding (GMAW). Plasma arc welding is also possible, but rarely utilized for WAAM of aluminum alloys. GTAW utilizes an inert tungsten electrode to establish an electric arc where the feedstock wire is introduced and transferred to the melt pool. The assembly with separate wire feeder and electrode complicates the deposition strategy of complex shapes [84]. On the other hand, several wires can easily be fed into the arc, increasing the deposition rate and enabling in-situ mixing of alloys. The latter is covered in more detail in Section 5.3. GTAW has a relatively high heat input [85], which may lead to bead overflow of the component [86].

An alternative GTAW setup was developed by Rodrigues et al. [87], termed ultracold WAAM (UC-WAAM). Unlike conventional deposition where the electric arc is established between the electrode and workpiece, UC-WAAM establishes an arc between the electrode and the feedstock wire. The short arc length provides a lower heat exposure to the already deposited component, leading to a smaller melt pool. A demonstrator case with carbon steel showed better forming possibilities and a higher cooling rate of the solidified material.

GMAW is more versatile for freeform fabrication, as the feedstock wire works as the electrode. The main volume of WAAM literature focused on the GMAW principle. From the early developments in the 1940s, GMAW saw continuous development into the 21st century. The most recent paradigm was set by the development of the cold metal transfer (CMT) principle in 2004 [88]. By short-circuiting the arc through dipping the filler wire into the melt pool, weld spatter was almost eliminated. Successful CMT of a range of aluminum alloys was demonstrated in recent years [25,36,89].

CMT was further developed with alternative deposition regimes in recent years. This includes a pulsing step (CMT-P) after short-circuiting [90], the possibility to exert alternating polarity of current (CMT-ADV) [28], and a combination of both (CMT-PADV) [23]. Comparative studies of the mentioned arc modes on aluminum alloys in terms of porosity and mechanical properties showed that the alternating current modes (CMT-ADV and CMT-PADV) created porosity-free structures with a fine microstructure [27,45].

Other amendments related to the hardware input for WAAM include shield gas composition. Shield gas mixtures with higher thermal conductivity (Ar-He) yield a deeper melt pool and higher cooling rates of the aluminum [91]. However, Ar-He shield gases have higher costs than that of pure Ar shield gases. N${}_{2}$ shield gas was shown to be unsuitable for WAAM of aluminum due to the formation of AlN nitrides, which reverted the mechanical properties [48]. The effect of oxygen impurities in the shield gas was explored by da Silva et al. [92], where an oxygen content below 200 ppm was found acceptable. Vibration of the substrate table was shown by Zhang et al. [93] to induce grain refinement and enhanced properties of Al-6Mg WAAM components. Heating of the feedstock wire prior to deposition was attempted to reduce porosity and increase production efficiency [30]. The importance in selection of substrate build material was highlighted by Eimer et al. [94].

Auxiliary control systems to ensure high-quality deposits are under continuous development. Algorithms for layer slicing and path planning prior to WAAM deposition is a research topic of considerable interest, as it may help with decreasing geometrical setoffs and increase complexity [95,96,97]. Heat accumulation during continuous deposition can deteriorate the WAAM design. Methods for in-line cooling of deposited material were examined, e.g., water immersion and air jet impinging [98,99,100].

In-situ process monitoring provides information of the process stability and detection of anomalies. Process information may be gathered by data from laser and IR sensors, weld signallers, pyrometers, ultrasound, spectrometers, and charge-coupled device (CCD) cameras to ensure the process quality [101,102,103]. Other hardware equipment include clamping fixtures to depress distortion, as well as in-situ or postmachining of the component to obtain a smooth outer- and inner-surface finish [104,105,106].

### 4.3. Microstructure Control

Physical and mechanical properties of metals are strongly related to their microstructure. The grain morphology and grain size influence the degree of anisotropy, strength, and ductility in aluminum alloys. A fine equiaxed grain structure was preferred for engineering applications, as the aforementioned properties are greatly enhanced. However, tight control of the microstructure evolution in melting-based processes such as casting and welding is challenging. For additive manufacturing with multiple melting-solidification cycles and steep thermal gradients, the situation is even more complex. The unique building strategy of AM can provide tailored spatial variations in the microstructure if the solidification behavior can be controlled [107].

**Figure 2:**
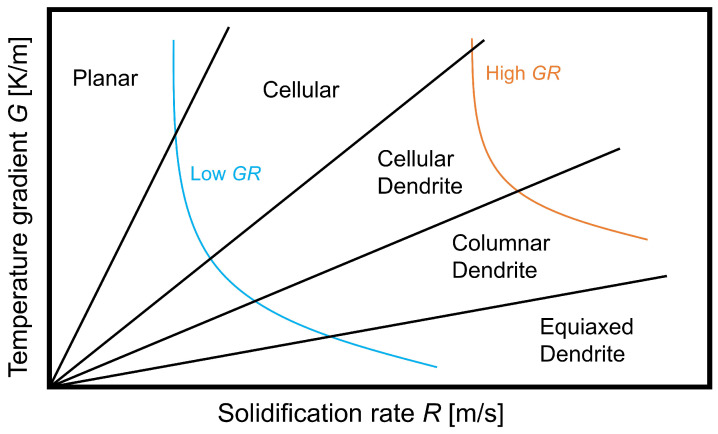
Effect of temperature gradient *G* and solidification rate *R* in the melt pool on solidification morphology and microstructural fineness. Reproduced with permission from Lippold; published by John Wiley & Sons, 2015 [110].

The grain morphology in the solidified structure is highly influenced by the heat conduction (i.e., *temperature gradient G* [K/m]) and the kinetics of mass transfer (i.e., *solidification rate R* [m/s]). The product GR [K/s], i.e., cooling rate, determines the microstructure fineness. The higher cooling rate, a finer structure is expected due to the decreased diffusion length during solidification. The G/R [$Ks/m^{2}$] ratio determines the interface morphology of the growing solidification front. Hence, the growth front stability is governed by G/R, which can be planar, cellular, or dendritic [108]. As seen in Figure 2 [109], equiaxed dendritic structures are formed at low G/R ratios. The most common grain structure in aluminum WAAM components is, however, columnar dendritic, due to the presence of a relatively high temperature gradient *G*.

The grain structure can be precisely tailored by controlling the G/R ratio. The principle was demonstrated by Dehoff et al. in powder-based AM for a nickel superalloy [111]. Columnar grains in Inconel alloys are highly textured along the <100> directions, while equiaxed grains are randomly oriented. Consequently, a tailored pattern can be created by the energy source. A similar approach was utilized by Yehorov et al. for WAAM of aluminum alloys [107].

**Figure 3:**
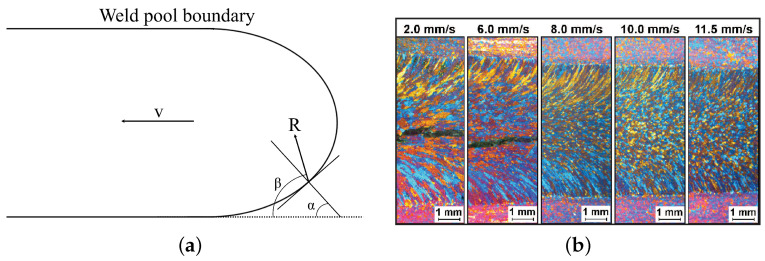
Effect of process parameters on grain morphology and cracking in aluminum arc welding. (**a**) Relationship between travel speed *v* and growth rate *R*. α is angle between torch travel direction and melt pool normal. β is angle between torch travel direction and actual solidification direction. Reproduced with permission from Kou; published by John Wiley & Sons, 2003 [85]. (**b**) Effect of travel speed *v* on solidification rate *R*, grain morphology, and cracking in arc welding of AA6082. An increased *v* induces equiaxed grain growth and suppresses cracking. Reproduced with permission from Schempp et al., Welding Journal; published by the American Welding Society, 2014 [114].

The solidification rate *R* of each individual grain is dependent on the scan speed *v* of the deposition torch in WAAM, as shown in Equation (4) [112]. The growth velocity is, however, uneven due to the nonuniform shape and heat conduction of the melt pool. This is accounted for by the angle α between the deposition direction and normal to the melt pool, as well as the angle β between deposition direction and actual solidification direction. These angles are shown in top-view for a melt pool in Figure 3a. The solidification rate is thus fastest parallel to the torch travel direction. The effect of torch speed *v* on the grain morphology of an AA6082 weld is shown in Figure 3b. Due to the increased *R* and corresponding decreased G/R ratio, the grain morphology in the center of the weld changed from dendritic columnar to dendritic equiaxed. Controlling the temperature gradient *G* can be obtained by increasing the heat input during deposition or by heating of the build substrate [113]. The input parameters must, however, be within the process window of stable WAAM deposition to avoid irremediable process faults.

(4) $$R=\frac{v\;cos\;\alpha }{cos(\alpha -\beta )}$$

### 4.4. Solidification in WAAM and CET

At present, only a fraction of the whole range of aluminum alloys is suited for WAAM. Issues regarding the cracking susceptibility during solidification excludes whole series of aluminum alloys. The limitations set by alloy selection establish a serious obstacle for industrial adaption. Strategies to avoid cracking need to be developed and exploited by metallurgists to present new alloys that are safe and reliable for use in WAAM.

As presented in Section 3.3, solidification cracking happens due to an increased pressure drop and insufficient back-feeding of liquid metal in a rigid solidified network. The rigid network is columnar in nature, which in certain cases (dependent on the solidification progress) establishes isolated melt pockets that cannot accommodate the shrinkage associated with final solidification. The melt distribution is highly increased when the solidification happens in an equiaxed manner instead. Combined with powerful grain refinement, the distribution distances are further decreased, leading to a significant reduction of the crack susceptibility. The effect of altering the grain growth mechanism is often termed columnar-to-equiaxed transition (CET), and it is one of the most powerful tools to reduce cracking and opens new alloys for WAAM. To understand the conditions and measures that lead to CET, the underlying concepts related to CET for WAAM are presented in the following paragraphs.

**Figure 4:**
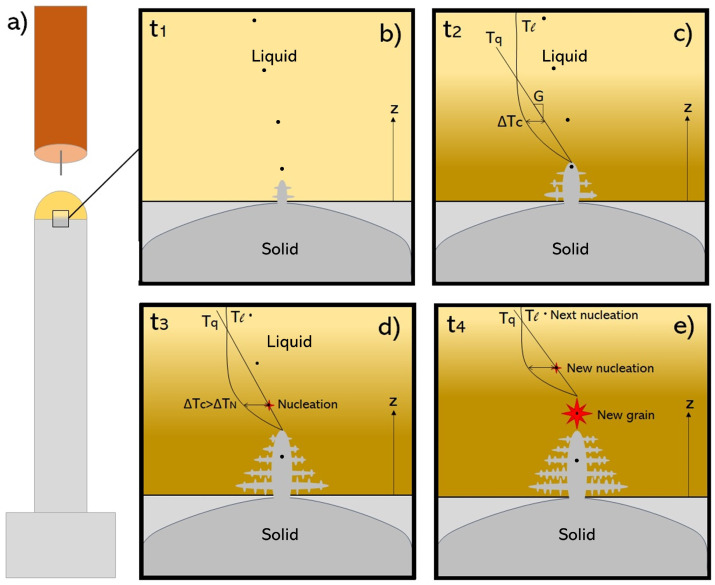
Schematic of epitaxial growth, development of a constitutional undercooled zone and heterogeneous nucleation during solidification of an alloy deposited by WAAM. (**a**) Illustration of a WAAM structure. Last layer is still in liquid phase (marked yellow). (**b**) Newly formed dendrite in the melt, epitaxially grown on the former layer. (**c**) Formation of a constitutional undercooled zone $\Delta T_{c}$ due to solute redistribution. Undercooling is insufficient for heterogeneous nucleation. (**d**) Heterogeneous nucleation of a new grain on a second-phase particle. (**e**) Growth of new grain and a new nucleation event. Reproduced with permission from Bermingham et al., Acta Materialia; published by Elsevier, 2019 [115].

Consider a WAAM setup with layer-wise deposition of aluminum by an arc torch, as shown in Figure 4a. The metal is transferred to the WAAM part as metal droplets that form a liquid melt pool. When the liquid starts to solidify, the lower part of the melt pool is in direct contact with the former layer. The former layer is in solid-state, and therefore holds a lower temperature. For aluminum alloys, heat dissipation by radiation to the surrounding atmosphere can be neglected. Heat is therefore conducted from the melt pool into the solid layer. Consequently, solidification starts at the solid–liquid interface between the former layer and the melt pool. The solid interface contains primary aluminum grains that are perfect heterogeneous nucleation sites due to the exact lattice match with aluminum. The melt starts to solidify on these grains, which is termed epitaxial growth, Figure 4b. The solidification continues where the dissipation of heat is fastest, i.e., in the opposite direction of the highest temperature gradient *G*, Equation (5). For the situation given in Figure 4c, the largest heat dissipation is vertically downwards towards the solid substrate. The high-thermal conduction of aluminum alloys sets up a strong heat sink towards the solid, i.e., a vertical *G* ($\left(\frac{\partial T}{\partial z}\right)>>\left(\frac{\partial T}{\partial x}\right)+\left(\frac{\partial T}{\partial y}\right)$). The combined contributions from epitaxial growth and directional heat sink stabilize the columnar dendritic structure.

(5) $$G=\left||\nabla T|\right|=\sqrt{{\left(\frac{\partial T}{\partial x}\right)}^{2}+{\left(\frac{\partial T}{\partial y}\right)}^{2}+{\left(\frac{\partial T}{\partial z}\right)}^{2}}$$

For alloys, the primary metal initially formed in the melt pool has a different composition to the bulk liquid. Solute is therefore redistributed into the melt, creating a solute-enriched diffusional zone ahead of the solidification front. The extension of this solute partition zone is dependent on the type and amount of alloying elements. The relation was equated by Desnain et al. in Equation (6) and termed growth restriction factor *Q* [116]. Each element *i* is regarded additive to each other if the solute is solved in the liquid. $m_{L}$ is the slope of the liquidus line, *k* is the partition coefficient (slope of the solidus line), and $C_{0}$ is the concentration of the alloying element. A list of *Q* values per wt.% for common solute elements in aluminum is given in Table 1. Note the high *Q* value of titanium, implying creation of a large partition zone.

(6) $$Q=\sum_{i=1}^{n}m_{L,i}\;\cdot \;\left(k_{i}-1\right)\;\cdot \;C_{0,i}$$

The transient solute-enriched diffusion zone has a lower equilibrium melting temperature $T_{l}$ than that of the bulk liquid in hypo-eutectic alloys. The real temperature $T_{q}$ in the solute redistribution region is, however, governed by the temperature gradient *G*. If the liquidus temperature gradient exceeds *G* (i.e., $\frac{dT_{l}}{dz}>G$), a so-called constitutionally undercooled zone is developed ahead of the solidification front, termed $\Delta T_{c}$ in Figure 4c. The constitutional supercooling $\Delta T_{c}$ stabilizes dendritic growth over planar growth [108]. As most aluminum WAAM structures exhibit a dendritic microstructure, constitutional supercooling ($\Delta T_{c}>0$) is present. The constitutional supercooling is thus the driving force for further growth of the columnar dendrite. The constitutional undercooled zone is promoted by increasing the growth restriction (i.e., *Q*), as given by Equation (7) [118]. $D_{L}$ is the diffusion coefficient of the solute in the liquid.

(7) $$\frac{G_{L}}{R}\geq \frac{-\sum_{i=1}^{n}m_{L,i}\;\cdot \;\left(k_{i}-1\right)\;\cdot \;C_{0,i}}{kD_{L}}=\frac{-Q}{kD_{L}}$$

If a solid surface (e.g., a particle) exist ahead on the solid/liquid interface as in Figure 4d, it can serve as a heterogeneous nucleation point for a new grain. The particle is activated as a nucleant if the constitutional supercooling $\Delta T_{c}$ surpasses the nuclei activation temperature $\Delta T_{N}$. In other words, heterogeneous nucleation on the particle is stable if $\Delta T_{c}>\Delta T_{N}$. A grain refining effect is thus established. If sufficient potent particles are available in the melt, repeated nucleation events take place in the melt pool, Figure 4e. The columnar growth is thus restricted, and the solidified structures appear as equiaxed dendritic [115].

Thus, CET needs two prerequisities to be effective in WAAM: a sufficiently large, undercooled zone ahead of the solid/liquid interface, and the presence of a nucleation agent. The constitutional supercooling $\Delta T_{c}$ is dependent on alloy addition (through the relation of *Q*) and temperature gradient *G* at the solid/liquid interface. $\Delta T_{c}$ increases with increasing *Q* and decreasing *G*. Potent nucleation agents have a low activation supercooling $\Delta T_{N}$, as heterogeneous nucleation takes place at $\Delta T_{c}>\Delta T_{N}$. $\Delta T_{N}$ is related to the surface energy that needs to be overcome for an aluminum atom to grow on the nucleant. Zhang et al. [119] proposed that a high degree of crystallographic matching between the two constituents determines the nucleation potency. CET is also a function of the nucleant density $N_{0}$, as shown in Equation (8) [120,121]. The maximum temperature gradient *G* to enable CET is increased by increasing $N_{0}$. Grain refinement is thus simplified by increasing the density of potent nucleation points. Furthermore, a low nucleant activation supercooling $\Delta T_{N}$ is beneficial for CET.

(8) $$G<0.617N_{0}^{1/3}\left(1-\frac{\Delta T_{N}^{3}}{\Delta T_{c}^{3}}\right)\Delta T_{c}$$

## 5. Aluminum Alloys for WAAM

### 5.1. Commercial Selection

Available feedstock wires for WAAM are today mostly ordinary welding wires. A supply network of wires tailor-made for WAAM is emerging [122,123], but is still immature for aluminum alloys due to low demand and low production volume [124,125]. The processes of arc welding and WAAM are fundamentally similar, and the wire selection is therefore identical at present. Aluminum alloys for welding are developed to meet a limited range of operations, e.g., joining of sheets and extrusions. The alloy selection is therefore limited to a few compositions, including the already mentioned 2319, 4043, 4047, 5087, 5183, and 5356 alloys. Other alloys available as feedstock wire but not demonstrated for WAAM include 1070, 1450, 5554, 5754, and 6063 [52,126].

WAAM has the potential to create aluminum components with intelligent design and superior mechanical properties. The basic prerequisite to obtain sound properties is closely related to the alloy composition. As the commercial alloy assortment is narrow at present, research and development is needed to expand the selection. This is essential for high-strength aluminum alloys belonging to the 2000, 6000, and 7000 series. Strategies and recent advancements of new alloys and compositions for WAAM are presented in the following.

### 5.2. Other Alloys for WAAM

The major restriction of new aluminum alloys for welding is the cracking susceptibility. As stated in Section 3.3, several alloys crack in the final stages of solidification due to contraction and an unfavorable microstructure. The contraction stresses are relatively high in traditional welding due to the constraint of the base metal (sheets, extrusions, etc.). For WAAM, only the former layer makes a mechanical constraint to the solidifying metal, and the shrinkage is easier to accommodate. With this in mind, a few attempts of WAAM with established alloys were demonstrated.

A comparative study by Haselhuhn et al. [127] involved the commercially pure 1100 alloy and the 4943 alloy. The 1100 alloy was as expected soft with relatively high ductility. The 4943 alloy with high silicon content (5%) and trace amounts magnesium (0.50%) exhibited no tendency of cracking, and was therefore successful for WAAM. The mechanical properties of 4943 were comparable to the 4047 Al-12Si alloy. Addition of magnesium to aluminum alloys with high silicon content enables the formation of the strengthening β phase. The Al-7Si-0.6Mg (4220) alloy is suitable for melting-based processing without tendency to crack [128]. Thus, these alloys are well-suited for WAAM combined with artificial aging [129,130]. Yang et al. showed that 4220 in as-deposited state has a relatively poor tensile strength of 130 MPa, but can be raised to 350 MPa in T6 state [131,132].

Preliminary studies on the applicability of the Al-Mg-Si alloy 6016 were performed by Ünsal et al. [133]. T6 treatment of the deposited material exhibited comparable strength to wrought material (238 MPa), however, with reduced ductility. The porosity content (<1%) and scattered cracking events were believed to cause the reduced ductility. Similar performance was reported by Hauser et al. for 6060 [134]. A few studies regarding established 2000-series alloys have been reported. Zhang et al. manufactured thin walls of the Al-Cu-Mg alloy 2024 with no tendency of cracking [135]. The beneficial composition of 2024 to suppress solidification cracking in WAAM was first reported by Fixter et al. [31]. The Al-Li alloys 2196 and 2050 with high strength-to-weight ratio was successfully deposited by WAAM [136,137]. Artificial T6 treatment promoted formation of T1 Al${}_{2}$CuLi precipitates, which accounted for a tensile strength reaching 439 MPa. The high vapor pressure of lithium makes Al-Li alloys challenging to deposit due to elemental losses and porosity formation.

### 5.3. Alloy Modifications

Hundreds of tailored aluminum alloys exist to meet specific end uses. The alloy composition is a fine-tuned balance between properties such as strength, ductility, extrudability, forgeability, machinability, weldability, among others. In this context, the adaption of already existing wrought alloys for WAAM is suboptimal given the fact that WAAM is a manufacturing route relying on fusion of the material. This contrasts with wrought alloys that are commonly formed in solid state. This effect can be seen on the weldability (i.e., tendency to crack) and resulting mechanical properties. Scientists and engineers should therefore focus on developing new alloy compositions tailored for WAAM.

The development of new alloy compositions for WAAM may be a tedious and resource demanding task. One approach utilized by several authors involves casting of a billet with a given composition, followed by hot rolling and drawing to wire dimensions. Then, the wire is deposited by WAAM, and the final properties are characterized. To examine a new or modified composition, the whole process chain must be repeated. Investigations of new compositions from the 7000 series [138,139,140,141], 2000 series [142,143], and 4000 series [144] using the casting route were reported in recent years. Experimental screening of potential aluminum alloys through casting is therefore regarded impractical and better suited for pilot experiments after screening.

An alternative method for screening potential alloy compositions is to mix different alloy additions directly in the arc plasma. The strategy of so-called twin-wire or multiwire WAAM emerged in recent years. The method involves multiple wire feeders leading feedstock of different compositions into a GTAW arc where the droplets instantaneously melt and mix. By changing the characteristics of each wire *i* (i.e., elemental composition $E_{x}$, diameter $D_{i}$, density $\rho_{i}$) coupled with the wire feeding rate $WFS_{i}$, the resulting element composition *E* can be precisely tailored. Qi et al. [145] mixed commercial 2319 and 5087 wires to obtain the 2024 alloy, previously seen to be highly suitable for WAAM. A similar approach to obtain 7050 WAAM deposits was demonstrated by Yu et al. [146] by combining 2319, 5356, and pure Zn wires. The crack-susceptibility of 7050 was profound, which limited the mechanical properties.

(9) $$E=\frac{\left(WFS_{i}D_{i}^{2}\rho_{i}E_{x}\right)}{\left(WFS_{i}D_{i}^{2}\rho_{i}\right)}$$

The fast screening through multiwire deposition can be utilized to find alloy composition especially suited WAAM with reduced cracking susceptibility, sound mechanical properties, and flowability, among others. An extensive screening of 27 different compositions of Al-Cu-Mg to evaluate the cracking tendency was performed by Gu et al. [16]. In fact, one half of the investigated alloys exhibited cracking post WAAM, highlighting the importance of alloy additions. Similar activities were performed by Klein et al. [147] and Qi et al. [148,149]. Eimer et al. [150] utilized laser assisted WAAM to mix pure zinc wires with 2319 to create high-zinc 7000 alloys.

### 5.4. Microalloying

Minor additions (<1%) of elements to the alloy chemistry to alter the microstructure and properties is termed microalloying. The art of microalloying was successfully implemented in steelmaking, developing a whole class of steels relying on small additions of Nb, V, Ti, Mo, Zr, and other elements to obtain sound mechanical properties and excellent weldability. Microalloying is also implemented in other material systems, such as nickel, titanium, and aluminum [151,152,153].

Microalloying elements are primarily used to refine the microstructure and enhance mechanical properties. The microstructural refinement is obtained through heterogeneous nucleation in the liquid-to-solid transition, and as obstacles for grain growth at elevated temperatures. Small microalloying particles exert a pinning pressure to counteract the driving force for a migrating grain boundary, often referred to as Zener pinning. Further, certain microalloying elements provide precipitation hardening by formation of semicoherent intermetallics.

A range of microalloying elements were demonstrated to enhance strength, fatigue, and creep resistance of aluminum alloys. This includes small additions of the transition metals Sc [154], Zr [155], Cd [156], Nb [157], Ti [158] and the rare-earth elements Ce [159], Hf [160], Yb [161], and Er [162]. Scandium and zirconium were of particular interest due to their grain refining effect. The excellent lattice match of the intermetallics Al${}_{3}$Sc, Al${}_{3}$Zr, and Al${}_{3}$(Zr${}_{1-x}$Sc${}_{x}$) lead to heterogeneous nucleation upon solidification of the aluminum alloy. Furthermore, the strength contribution of these phases are prominent due to the formation of tiny L1${}_{2}$ precipitates through heat treatment [163,164,165].

Microalloying of the wire chemistry for arc fusion was demonstrated numerous times in the literature. Traditional fusion welding with 7000-alloys was enabled with additions of Sc and Zr [166,167]. CET and the related grain refinement suppressed the cracking susceptibility. For WAAM, Sc was of particular interest and added to various 5000-series alloys [168,169,170]. The rapid solidification in WAAM supersaturated the Sc addition leading to significant precipitation strengthening post-WAAM [171]. Zr is often used as a substitute due to the high cost of Sc. The use of combined additions of Sc and Zr to an Al-6Mg alloy for WAAM was recently demonstrated by Ponomareva et al. [172]. At optimum aging conditions after WAAM, a high-strength material with ultimate tensile strength of 408 MPa was developed.

Titanium creates the Al${}_{3}$Ti phase with aluminum which is regarded as a highly potent nucleation site [119]. Titanium in solid solution also provide strong growth restriction of the solid–liquid growth front during solidification, creating a large constitutional undercooling. Both effects are important to refine the grain structure. This was utilized by Wang et al. for deposition of the 5356 alloy in WAAM [173]. A titanium suspension was sprayed on the hot aluminum metal after deposition, letting the organic suspension evaporate before a new layer was made. The addition of Ti induced CET, and the average grain size was reduced. Titanium does not provide precipitation strengthening in aluminum. The strength enhancement was therefore modest, with an increase in tensile strength from 253 MPa to 273 MPa.

Microalloying with titanium in high-silicon aluminum alloys is inefficient for grain refinement. Titanium has a stronger affinity to silicon than aluminum, leading to the formation of ternary Al-Si-Ti intermetallics. The grain refining effect of Al${}_{3}$Ti is thus not utilized in high-silicon alloys from the 4000 series. This effect was experienced by Li et al. [138] for WAAM deposition of an Al-7Si-0.6Mg alloy with up to 0.3 wt.% Ti. The base alloy chemistry must therefore be considered upon microalloying.

Niobium provides a similar effect as Ti in terms of grain refinement. The formation of the Al${}_{3}$Nb phase significantly refined the WAAM microstructure of an Al-6Mg alloy [174]. A coarse columnar was changed to a fine equiaxed structure, which increased the ultimate tensile strength by 58 MPa. As Nb has little-to-no reactivity with Si, it works as a substitute for Ti in high-silicon alloys [175]. Hypoeutectic additions of tin (<0.12 wt.% [176]) showed the ability to grain refine Al-Cu alloys and increase the density of the strengthening $\theta^{\prime }$ phase. A combination of enhanced tensile strength and elongation was demonstrated with proper WAAM parameter control [177,178].

### 5.5. Ceramic Particle Additions

Another strategy to enhance the performance of aluminum alloys for WAAM is addition of a second phase material. By combining two or more materials with significantly different properties, the resulting mixture exhibits characteristics from each individual phase, e.g., the ductility of a metal and the hardness of a ceramic.

The driving forces of ceramic additions to aluminum alloys for additive manufacturing are microstructural refinement and strength enhancement. Several ceramic compounds show a grain refining effect in aluminum through heterogeneous nucleation. TiB${}_{2}$ has been used as grain refiner for aluminum ingot casting for decades. The transmission of refining welds with TiB${}_{2}$ did not find industrial application, although it was demonstrated to be efficient at restricting solidification cracking [114]. The solution is directly transferable to WAAM [179,180]. The use of the less costly TiC particles was also of interest for arc welding [181] and WAAM [182]. In fact, the altered solidification progress of the Al-Cu 2219 alloy by addition of TiC hindered solute segregation of Cu on grain boundaries, keeping the atoms in solid solution after WAAM. The combined effects of hard TiC particles and enhanced solid solution strengthening raised the tensile strength from 263 MPa to 403 MPa [182].

The grain refinement by addition of ceramic particles induces CET during solidification in WAAM. The equiaxed grain morphology is more tolerant to shrinkage stresses compared to the elongated columnar counterpart. CET thereby suppresses the cracking tendency experienced by a range of aluminum alloys. Hard-to-deposit alloys can therefore be made available for WAAM, as shown for 6063 [183] and 7075 [184].

The emerge of nanoparticle additions yield significant strength contributions to the final material if the nanoparticles are finely dispersed in the aluminum matrix. Nanoparticles are nonshearable for dislocations, which pin the dislocations and create Orowan loops (bowing dislocations) around the particles. The increased dislocation density hardens the material by the so-called Orowan strengthening mechanism. In addition, geometrically necessary dislocations are generated due to the difference in coefficient of thermal expansion (CTE) between aluminum and nanoparticles upon cooling. These effects were demonstrated by the use of nanosized (<100 nm) TiC in arc welding [185,186]. Other ceramic phases are believed to contribute in a similar manner to enhance the strength provided a small particle diameter and fine dispersion in the aluminum matrix.

Viable processing routes are an important prerequisite for implementation of ceramic reinforcements in aluminum alloys. Aluminum mixed with ceramics are manufactured through a range of processing routes, including thermal spraying and electrochemical deposition, but more commonly through powder metallurgy and stir casting principles. Powder metallurgy routes are highly suited for powder-based additive manufacturing, and several examples of Selective Laser Melting (SLM) with ceramic-reinforced aluminum are demonstrated in the literature [187,188,189,190]. Composite wire production for arc or laser deposition are commonly obtained through casting principles. The reinforcement phase is commonly a ceramic with high melting point, and hence, present in solid-state in the aluminum cast liquid. Settling and agglomeration of the reinforcement phase is therefore a common challenge, and methods to agitate the aluminum melt are often necessary. This is commonly achieved by mechanical stirring with rotor blades or ultrasonic vibration of the crucible. Another consideration is the wettability of the reinforcement phase with aluminum; if the reinforcements have a high interface energy towards the liquid, it will agglomerate to reduce the total surface energy. The task becomes even more difficult with decreasing particle size; nanoparticle additions are therefore considered challenging to disperse by stir casting [191]. A workaround of this challenge was the use of fluxing agents to make nanoparticles comfortable in the melt. This was demonstrated for TiC nanoparticles by Liu et al. [192], where potassium tetrafluoaluminate (KAlF${}_{4}$) was used as fluxing agent. Another consideration is the reactivity of the reinforcement with the matrix melt, which greatly limits the selection of type of additives to aluminum alloys. The aforementioned reactivity between Ti and Si and the instability of carbon nanotubes in aluminum melts are some examples [193,194].

To avoid the mentioned challenges related to casting, Langelandsvik et al. examined a new solid-state route for production of feedstock wires for WAAM. Based on the metal screw extrusion principle [195], fragmented aluminum pieces were mixed with ceramic nanoparticle powder into a ’mincer’ driven by an Archimedes screw. The individual fragments were consolidated to a single volume, compressed, and extruded as a wire [196]. The torsional component of the screw motion dispersed the nanosized reinforcement phase without excessive agglomeration. Addition of TiC nanoparticles to the wire resulted in efficient grain refinement of the 5183 alloy [197].

Casting and extrusion principles rely on incorporation of the reinforcement phase into the matrix material. However, issues regarding agglomeration, settlement, and chemical instability may revert the performance of the reinforcements. The costs of screening different alloy-reinforcement combinations are also unacceptably high. Methods to avoid the relatively costly production routes for aluminum mixed with secondary phases are thus beneficial. A solution presented by several works involves coating the WAAM material with the reinforcement phase dispersed in an organic suspension [180,198]. The suspension is sprayed on the WAAM layer while it is still hot to obtain accelerated evaporation of the organic species. The remnants are then incorporated into the melt pool when a new layer is deposited. The electromagnetic stirring of the melt pool ensures a good mixture of the reinforcement. Microcasting is another rapid process route to examine a wide range of material combinations. The arc melting method is well-suited for such applications, where a small powder addition is fused by an electrical arc in a controlled atmosphere. The lead time from sample preparation to finished sample is short, and hence, is well-suited for screening. With optimized process parameters, arc melting can mimic the thermal cycle of WAAM and provide an insight of the resulting properties of the composite mixture.

## 6. Future Developments

Every aspect of the WAAM process needs further development to lower the threshold of widespread industrial acceptance. WAAM is a disruptive technology, where increment-based fusion and solidification of material is used to create a structure. This stands in contrast to traditional manufacturing, which relies of solid-state forming, such as forging or machining, or liquid-state forming such as casting. The materials and alloys available for wire based additive manufacturing today are to a large degree designed for traditional fusion-based welding and brazing. Materials tailored for WAAM are highly sought for the development of a future additive industrial environment.

Each WAAM component needs to meet the specific properties for its end use, e.g., tensile strength, stiffness, fatigue, or corrosion resistance, etc. The properties are related to the microstructure, which is highly connected to the alloy composition. The chemical composition of the feedstock wire must be tailored to yield the desired microstructure in the end component. Several aspects are relevant in this respect.

The effect of grain morphology was pointed out in this review. Promotion of an equiaxed, fine-grained structure is regarded as the single most important measure to avoid the formation of solidification cracks, which pester several aluminum alloys. Equiaxed grains also lower the anisotropy of the structure and can improve the mechanical properties. Measures to effectively achieve desired grain structures were pointed out and need to be implemented into future feedstock wires for WAAM. Of vital importance is the work related to expanding the alloy selection including high-strength systems of the 2xxx-, 6xxx-, and 7xxx-series.

Deposition of feedstock wire by an electric arc implies a high-temperature melting cycle of the aluminum alloy. Element losses of volatile elements are well-documented for WAAM of aluminum, i.e., Mg, Zn and Li [58,136]. The degree of evaporation during the process must be well-understood, and an ’overalloying’ strategy could be developed to account for the element loss. Element vaporization favors the use of low-energy deposition torches.

As the feedstock wire is the ’building block’ in WAAM, the wire quality is of utmost importance. The outer characteristics can highly influence the material quality after deposition. The wire diameter needs to be even without fluctuations to avoid arc instabilities. The wire surface must be smooth without dimples and scratches. Perturbations on the wire surface is a trap site for grit and moisture during storage and handling. In general, the impurity levels, especially hydrogen, must be kept to an absolute minimum to avoid the formation of porosity.

WAAM is projected to become a vital contributor in the aerospace and aeronautical industries. The safety regulations in these sectors are strict, with high demand for documentation and certification. Time and resources must be allocated for certification of new and promising aluminum alloys suited for aerospace applications.

## 7. Conclusions

WAAM is a promising manufacturing route for the next industrial generation. The high deposition rate, low investment costs, and versatile application area make the process highly suited for component production, repair, and refurbishment. WAAM of aluminum alloys needs to overcome several metallurgical and structural challenges to raise the material quality and attractiveness of the industry. This includes porosity formation, residual stress generation, and crack formation. This review provided an insight into these WAAM-related defects and presented viable solutions to overcome these obstacles. Special attention was devoted to the alloy selection. A survey shows that nearly a dozen aluminum alloys are commercially available as WAAM feedstock. A majority of these materials are manufactured for traditional arc welding and are not tailored for WAAM. A separate supply chain for WAAM feedstock is emerging, but is at present experiencing a scarcity of aluminum. The adoption of WAAM into service is unattractive when desired micostructures and properties are unattainable. Thus, future research and development focusing on new aluminum alloys tailored for WAAM should be prioritized. Strategies to combat the formation of cracking commonly seen in several aluminum alloys were reviewed. The importance of alloy composition and grain structure was of particular interest. By altering the grain morphology from columnar to equiaxed, the cracking susceptibility is greatly reduced. Screening methods to develop new ’WAAMable’ aluminum alloys were described. Modeling of solidification progress, multiwire WAAM, and arc melting are three efficient strategies for alloy development. The possibility of aluminum WAAM materials with desired properties wide-spread industrial operation is attainable by proper implementation of these strategies.

## Figures and Tables

**Table 1:** Growth restriction factor *Q* of elements in solution with aluminum. Input data are based on binary phase diagrams [117].

Element *i*	$k_{i}$	$m_{L,i}$	$Q/C_{0,i}$
Si	0.11	−6.6	5.9
Mg	0.51	−6.2	3.0
Mn	0.94	−1.6	0.1
Cu	0.17	−3.4	2.8
Zn	0.88	−2.97	0.3
Fe	0.02	−3.0	2.9
Ti	7.8	33.3	220
V	4.0	10.0	30
Mo	2.5	5.0	7.5
Nb	1.5	13.3	6.6
Cr	2.0	3.5	3.5
B	0.067	1.0	3.2

## Data Availability

The data presented in this study are available on request from the corresponding author.

## Abbreviations

The following abbreviations are used in this manuscript:

AM	Additive manufacturing
ASTM	American Society for Testing and Materials
CCD	Charge-coupled device
CET	Columnar-equiaxed-transition
CNC	Computer numerical control
CMT	Cold Metal Transfer
CMT-ADV	Cold Metal Transfer Advanced
CMT-P	Cold Metal Transfer Pulse
CMT-PADV	Cold Metal Transfer Pulse Advanced
CTE	Coefficient of Thermal Expansion
FCC	Face-centered cubic
GMAW	Gas metal arc welding
GTAW	Gas tungsten arc welding
LCA	Life-cycle assessment
RDG	Rappaz–Drezet–Gremaud
SLM	Selective Laser Melting
WAAM	Wire arc additive manufacturing
